# Fahr’s Syndrome Presenting As Pre-senile Dementia With Behavioral Abnormalities: A Rare Case Report

**DOI:** 10.7759/cureus.20680

**Published:** 2021-12-25

**Authors:** Ajinkya S Ghogare, Shyam Nemade

**Affiliations:** 1 Psychiatry, Manoday Mansopchar Clinic, Akot, IND; 2 Pathology, Dr. Nemade's Nidan Pathology Laboratory and Research Center, Akot, IND

**Keywords:** fahr's disease, hypo-parathyroidism., pre-senile dementia, basal ganglia, fahr’s syndrome

## Abstract

Fahr’s syndrome is a rare neurological disorder characterized by bilateral basal ganglia calcification. Calcification may also involve other brain areas like dentate nuclei of the cerebellum, thalamus, cerebral cortex, hippocampus, and subcortical white matter. Many cases of Fahr’s syndrome present with movement disorders, but may also present with dementia, psychiatric manifestations, and language difficulties. Fahr’s syndrome generally occurs secondary to metabolic abnormality mainly hypoparathyroidism. Fahr’s disease is another variant that is characterized by idiopathic bilateral calcification of basal ganglia in absence of any evident etiology. The present case report presented a rare case of Fahr’s syndrome secondary to hypoparathyroidism presenting with pre-senile dementia with behavioral abnormalities.

## Introduction

Fahr’s phenomenon is a rare neurological disorder occurring in less than 1 per 1,000,000 populations [1,2]. It has two variants namely Fahr’s syndrome and Fahr’s disease. The signs and symptoms of Fahr’s syndrome and Fahr’s disease resemble one other, except for the etiology. Fahr’s syndrome is always secondary to the underlying etiology of which the most common is hypoparathyroidism, while Fahr’s disease is idiopathic in nature [3]. Age of onset also differs between the two conditions. Fahr’s syndrome generally occurs among individuals between 30 and 40 years of age, while Fahr’s disease occurs among individuals between 40 to 60 years of age [3].

It has various manifestations such as movement abnormalities (in the form of tremors, chorea, and Parkinsonism), dementia, cerebellar dysfunctions, and neuro-psychiatric presentations like personality changes, behavioral abnormalities, psychosis, depression, and worsening of intellectual functioning [1,2,4-8].

Fahr’s syndrome is characterized by the bilateral symmetrical dense calcification of basal ganglia. There is abnormal calcium deposition in various brain areas such as basal ganglia, dentate nuclei of the cerebellum, thalamus, cerebral cortex, hippocampus, and subcortical white matter [9].

The most common etiology of Fahr’s syndrome is hypoparathyroidism [2]. Computed tomography (CT) of the brain is a preferred modality to locate and assess the extent of cerebral calcification [2]. Other basic tests used to know the etiology include serum concentrations of calcium, magnesium, phosphorus, calcitonin, alkaline phosphatase, and parathormone (PTH) [2]. Ellsworth-Howard test in which stimulation with 200 micromoles parathyroid hormone (PTH) leads to increased excretion of cyclic adenosine monophosphate (cAMP) in urine by 10 to 20 folds helps in diagnosis of Fahr’s syndrome [2]. Few studies have indicated the importance of measuring vitamin D3 levels [10,11]. Natural killer (NK) cells concentration must be assessed in all cases as it was found to be raised in patients with Fahr’s syndrome [12]. Mini-mental state examination (MMSE) is a tool used for categorizing the severity of cognitive impairment among patients with dementia. MMSE scores of 0 to 10, 11 to 19, 20 to 24, and 25 to 30 indicate severe, moderate, mild, and questionably significant cognitive impairment [13].

## Case presentation

**Figure 1:**
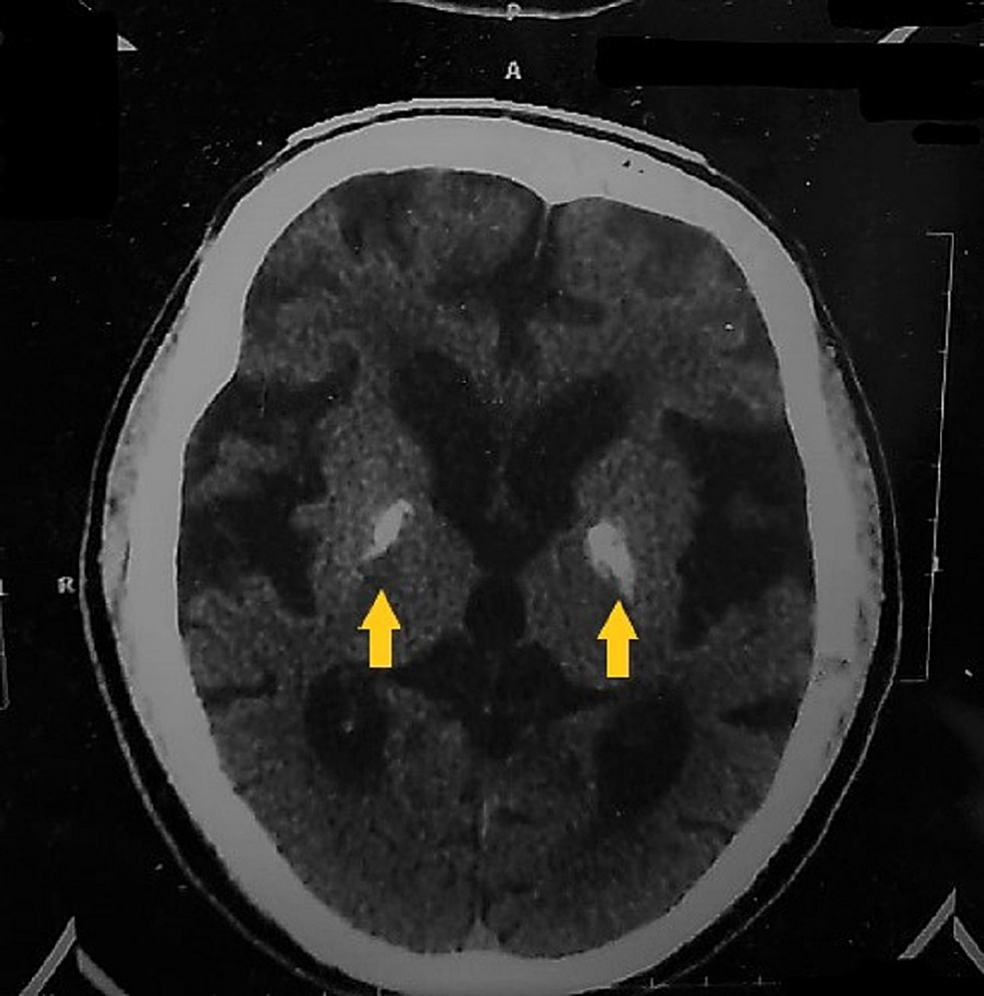
CT Brain showing bilateral basal ganglia whitish calcification (indicated by the colored arrows).

**Figure 2:**
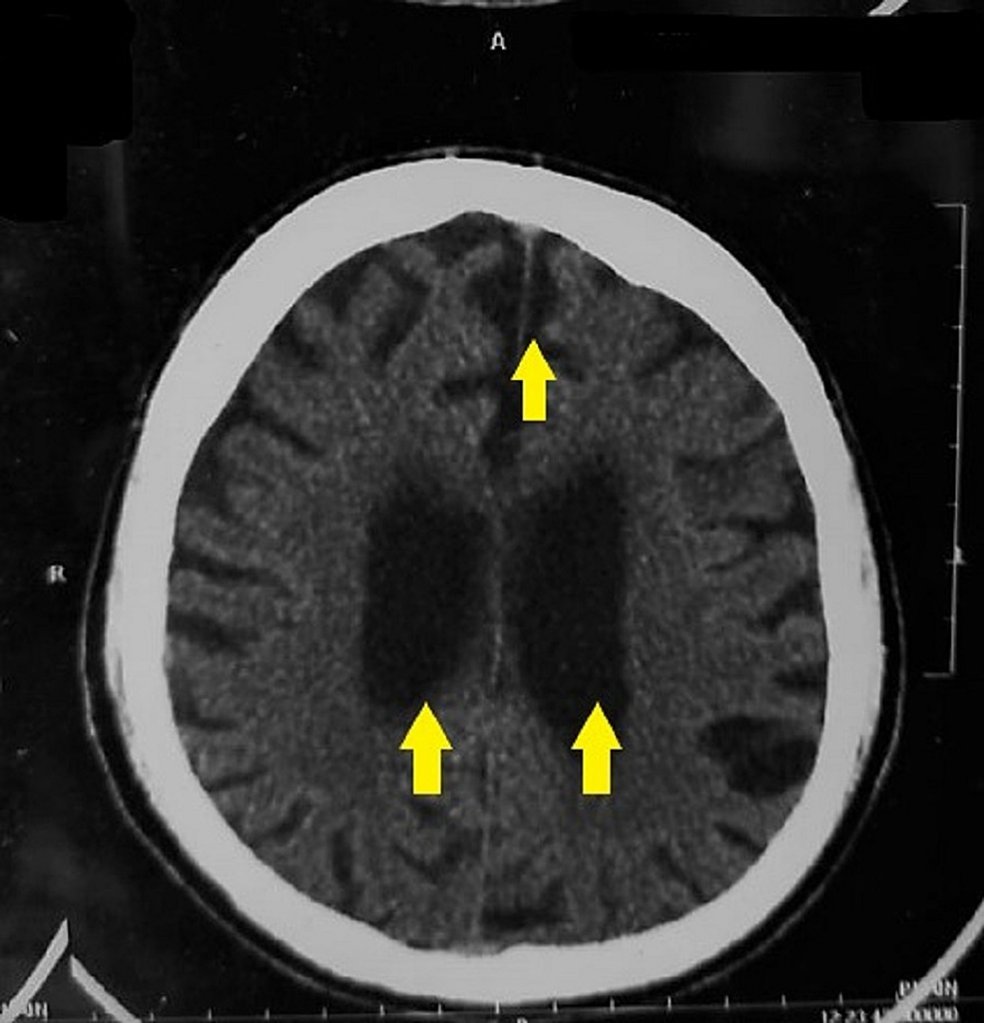
CT Brain showing bilateral cerebral atrophy in the form of enlarged cerebral ventricles and widened sulci (indicated by the colored arrows).

A 38-years-old separated male, living with his mother, presented to the psychiatric outpatient clinic along with his mother with the primary complaint of progressive forgetfulness since six years. According to the mother, the patient had gradual deterioration of his cognitive abilities like not being able to remember all the recent events and most of the past events, not being able to recognize most of the relatives and their names, not being able to read and write in Marathi, Hindi, and English languages that were once learned by him, not being able to handle finances, not being able to do works related to his daily routine as well as farm-related work. He became totally dependent on his wife and mother for everyday activities including his own hygiene. Gradually he started talking irrelevantly and because of all his deterioration his spouse allegedly left him about two years back. He underwent a CT scan of the brain three years back in which it was shown that he had bilateral dense basal ganglia calcification and cerebral atrophy (Figures 1-2).

Prior to his first visit to the treating psychiatrist, he already received treatment from two neurologists in the form of oral donepezil in varying doses that ranged from 5mg to 15mg per day, but the compliance on the patient’s and caretaker’s side was poor with minimal symptomatic improvement.

He was having sleep disturbances for the past twelve months. Since last three months he was exhibiting behavioral abnormalities like irritability, stubbornness, and crying episodes. He also had two episodes of soiling of clothes with feces and one episode of soiling of clothes with urine during the daytime when he was in awake state about two months back. Because his CT findings pointed towards the diagnosis of Fahr’s phenomenon and pre-senile dementia, basic blood investigations were done, which are presented in Table 1.

**Table 1:** Blood investigations of the patient PTH: Parathyroid hormone; TSH: Thyroid stimulating hormone

Blood parameter/investigation	Values in index patient	Normal/reference values
Serum PTH-intact (pg/mL)	10.80	14.00 to 72.00
Serum Calcium (mg/dl)	10.0	8.6 to 10.3
Serum Magnesium (mg/dl)	2.39	1.6 to 2.5
Serum Phosphorus (mg/dl)	4.38	2.7 to 4.7
Alkaline phosphatase (ALP) (U/L)	100	30 to 140
T3 – Total (ng/ml)	1.82	0.7 to 2.13
T4 – Total (μg/dl)	10.72	4.5 to 12.5
TSH – Ultrasensitive (μIU/mL)	1.75	0.34 to 5.6

Except for serum PTH concentration, which was low, all other investigations were normal. Based on the findings from CT Brain and blood investigations, which showed hypoparathyroidism, a diagnosis of Fahr’s syndrome with pre-senile dementia was made. His age at onset of the illness and underlying hypoparathyroidism were in favor of the diagnosis of Fahr’s syndrome. There was no family history suggestive of dementia and Fahr's syndrome. There was no history of movement disorder in the patient. On neurological examination, there were no signs suggestive of movement disorders, Parkinsonism, or any other major neurological conditions. His MMSE score was 9, which was suggestive of severe cognitive impairment. He was prescribed oral donepezil and memantine (10mg+10mg) combination once a day, along with oral clonazepam 0.25mg at bedtime, oral sodium valproate 300mg twice a day, and oral multivitamins including 1500mcg methylcobalamin. The endocrinologist’s opinion was sought in view of hypoparathyroidism as it is the most common cause of Fahr’s syndrome, who prescribed oral Vitamin D3 in a dose of 200IU per day. On subsequent visits after the initial assessment, the dose of oral donepezil and memantine (10mg+10mg) combination was increased to twice a day for betterment in cognition, oral clonazepam was increased to 0.5mg at bedtime for the betterment of sleep, and oral sodium valproate was increased to 500mg twice a day for the betterment of behavioral abnormalities. During the subsequent follow-up visits his MMSE score was 11, which was slightly better compared to earlier and indicated moderate cognitive impairment, i.e. showed improvement in patient’s cognition from severe cognitive impairment to moderate cognitive impairment. He also showed a mild degree of improvement in his behavior and sleep while on treatment as per the history from his mother. During the course of treatment, there was slow but gradual improvement in the patient but unfortunately, he was lost during follow-up as his mother didn’t bring him for the regular consultations.

## Discussion

This case portrayed a rare case of Fahr’s syndrome which was secondary to hypoparathyroidism. This case confirms the findings of the study by Saleem et al. that the most common etiology of Fahr’s syndrome is hypoparathyroidism [2]. The CT brain of the index patient showed bilateral dense basal ganglia calcification. This was similar to the findings of the study by Ahad et al. that the most common site of the brain that gets involved in Fahr’s syndrome was basal ganglia [9]. The present study and earlier studies proved the notion that the presence of bilateral symmetrical basal ganglia calcification along with metabolic abnormality like hypoparathyroidism points towards the diagnosis of Fahr’s syndrome [2,14,15]. In our patient, there was no history of abnormal movements or Parkinsonism despite the involvement of basal ganglia. Another study observed that Fahr’s phenomenon was associated with Parkinsonian features, which improved on treatment with levodopa [16]. In our patient, dementia with behavioral abnormality was the main neuro-psychiatric presentation. A similar finding was noted by other studies also [15,17]. Non-familial and familial types of Fahr’s syndrome have been described in the literature [18]. Because of financial constraints, CT scans of the brain of the patient’s mother and sister were not possible for the detection of a familial form of Fahr’s syndrome and both were asymptomatic. Also, because of financial constraints, other investigations such as MRI brain, MR spectroscopy, and CT scans of the chest as well as the abdomen of the patient were not performed. Although the index patient didn’t follow up regularly for a longer duration, he had some improvement in cognition as evident on MMSE scores owing to the treatment with donepezil and memantine combination. Similar improvement was noted by another study where a patient with Fahr’s disease and dementia showed improvement on treatment with donepezil [19].

In the present study, the patient had severe cognitive impairment at the time of presentation based on the MMSE score. Rissardo et al. found that, based on the MMSE score, their study participant had mild cognitive impairment [20]. In contrast to the present case study, Rissardo et al. found that their study participant had movement disorder in the form of resting tremors in the right upper limb, and the postural and intentional tremors in the left upper limb [20]. Similar to the present study finding, Rissardo et al. also observed behavioral abnormalities in their study participant. In their study participant, the main behavioral abnormalities were changes in humor, sadness, and irritability [20]. The findings of behavioral abnormalities in the present case study and that of Rissardo et al. [20] point towards the underlying cause of cognitive impairment secondary to Fahr’s phenomena.

## Conclusions

Although rare, Fahr’s syndrome is commonly associated with various neuropsychiatric presentations and timely diagnosis of Fahr’s syndrome and its associated neuropsychiatric conditions like pre-senile dementia and behavioral abnormalities warrant attention to improve the outcome. For a better treatment outcome, consultation liaison should involve a team of psychiatrists, neurologists, endocrinologists as well as a psychiatric social worker who can provide required support to the suffering individual and his/her family members.

## References

[1] Ghogare AS, Agrawal SR, Patil PS (2020). Obsessive compulsive disorder with psychotic features as neuropsychiatric manifestation of Fahr's disease. Ann Indian Psychiatry.

[2] Saleem S, Aslam HM, Anwar M, Anwar S, Saleem M, Saleem A, Rehmani MA (2013). Fahr's syndrome: literature review of current evidence. Orphanet J Rare Dis.

[3] Perugula ML, Lippmann S (2016). Fahr’s disease or Fahr’s syndrome?. Innov Clin Neurosci.

[4] Taxer F, Haller R, König P (1986). Clinical early symptoms and CT findings in Fahr syndrome [Article in German]. Nervenarzt.

[5] Shouyama M, Kitabata Y, Kaku T, Shinosaki K (2005). Evaluation of regional cerebral blood flow in Fahr disease with schizophrenia-like psychosis: a case report. AJNR Am J Neuroradiol.

[6] Broncel M, Koziróg M, Zabielska J, Poliwczak AR (2010). Recurrent syncope and hypocalcaemic cardiomyopathy as manifestations of Fahr's syndrome. Arch Med Sci.

[7] Seidler GH (1985). [Psychiatric and psychological aspects of Fahr syndrome]. Psychiatr Prax.

[8] Baptista MV, Vale J, Leitão O (1997). Striato-pallido-dentate calcifications [Article in Portuguese]. Acta Med Port.

[9] Ahad MA, Kamal SM, Salma U (2017). Fahr’s syndrome: a rare neurodegenerative disorder. Med.

[10] Boller F, Boller M, Gilbert J (1977). Familial idiopathic cerebral calcifications. J Neurol Neurosurg Psychiatry.

[11] Morita M, Tsuge I, Matsuoka H, Ito Y, Itosu T, Yamamoto M, Morishima T (1998). Calcification in the basal ganglia with chronic active Epstein-Barr virus infection. Neurology.

[12] Morishima T, Morita M, Kato T, Hoshino Y, Kimura H (2002). Natural killer cell proliferation and circulating cytokines in patients with bilateral basal ganglia calcification. Eur J Neurol.

[13] Folstein MF, Folstein SE, McHugh PR (1975). “Mini‑mental state”: a practical method for grading the cognitive state of patients for the clinician. J Psychiatr Res.

[14] Oliveira JR, Spiteri E, Sobrido MJ (2004). Genetic heterogeneity in familial idiopathic basal ganglia calcification (Fahr disease). Neurology.

[15] Modrego PJ, Mojonero J, Serrano M, Fayed N (2005). Fahr's syndrome presenting with pure and progressive presenile dementia. Neurol Sci.

[16] Otu AA, Anikwe JC, Cocker D (2015). Fahr's disease: a rare neurological presentation in a tropical setting. Clin Case Rep.

[17] Cartier L, Passig C, Gormaz A, López J (2002). Neuropsychological and neurophysiological features of Fahr's disease [Article in Spanish]. Rev Med Chil.

[18] Kobari M, Nogawa S, Sugimoto Y, Fukuuchi Y (1997). Familial idiopathic brain calcification with autosomal dominant inheritance. Neurology.

[19] Jensen MP, Spasic-Boskovic O, Rowe JB, Galton C, Allinson KS (2020). Clinicopathological co-occurrence of Fahr's disease and dementia with Lewy bodies. Clin Neuropathol.

[20] Rissardo JP, Caprara AL, Silveira JO (2019). Fahr’s disease presenting with pure dementia: a case report and literature review. Apollo Med.

